# Design Optimization of RF MEMS-Driven Triangular Resonators with Sierpinski Geometry for Dual-Band Applications

**DOI:** 10.3390/mi16040446

**Published:** 2025-04-09

**Authors:** Alina Cismaru, Flavio Giacomozzi, Mircea Pasteanu, Romolo Marcelli

**Affiliations:** 1 National Institute for Research and Development in Microtechnologies, 077190 Bucharest, Romania; alina.cismaru@imt.ro (A.C.); mircea.pasteanu@imt.ro (M.P.); 2 Fondazione Bruno Kessler (FBK), 38123 Povo, TN, Italy; giaco@fbk.eu; 3 Consiglio Nazionale delle Ricerche (CNR)-Institute for Microelectronics and Microsystems (IMM), 00133 Roma, RM, Italy

**Keywords:** RF MEMS, SPDT, microwaves, dual-band operation, triangular resonators, Sierpinski geometry

## Abstract

This paper proposes a detailed design study of resonating high-frequency notch filters driven by RF MEMS switches and their optimization for dual-band operation in the X-Band. Microstrip configurations will be considered for single and dual-band applications. An SPDT (single-pole-double-thru) switch composed of double-clamped ohmic microswitches has been introduced to connect triangular resonators with Sierpinski geometry, symmetrically placed with respect to a microstrip line to obtain a dual notch response. Close frequencies or spans as wide as 2 GHz can be obtained depending on the internal complexity and the edge side. The internal complexity has been modified to introduce the possibility of using the same edge size for the frequency tuning of an elementary cell, maintaining a fixed footprint, and allowing coupled structures to implement high-frequency filters of the same size and variable operational frequencies. Preliminary experimental results have been obtained as a confirmation of the predicted device functionality.

## 1. Introduction

The utilization of fractal geometries for high-frequency signal processing has been a hot topic for a couple of decades, aiming to provide a multi-band response with reduced size and increasing geometrical complexity, either in planar or volume configurations. A review for antenna purposes is given by [1]. Often, these structures are inspired by a metamaterial design and the tunability is given by switching single components of the structure [2]. Moreover, by changing the fractal structure’s complexity and the figure’s planar extension, high-frequency applications can benefit from frequency tuning due to both characteristics. RF MEMS switches introduce a further advantage in the tuning performance, addressing the signal to routes characterized by different operative frequencies [3,4,5]. The above solution has been studied by combining single microswitches in a single-pole-double-thru (SPDT) structure to obtain devices in coplanar waveguide (CPW) configurations with CPW-based notch filters driven by RF MEMS SPDT switches [6]. Sierpinski-inspired triangular resonators with a CPW arrangement have also been studied in [7]. The advantages of the symmetric configuration of the resonators along the feeding line have been outlined, as well as the general benefits in terms of quality factor, footprint, and operational frequency increase due to the triangular shape, which differ compared to those achieved for square and circular geometries [8]. The above findings have been inspired by the original work described in [9,10], where the spectrum of the first observable modes was studied according to analytical formulations and experimental results. That study outlined that the triangular shape of the resonators favors lower radiation loss and higher unloaded quality factors. The seminal results about the advantages of the triangular shape were stressed when using waveguide arrangements in Y-circulators in both cited papers.

In this contribution, a microstrip configuration has been chosen for planar triangular resonators as an alternative to the already studied CPW-fed ones, with the advantage of a less critical design because the role played by the lateral ground plane is now absent. This result was obtained owing to the simplification of the boundary conditions. Choosing an optimized distance between the resonator and feeding line that can also be imposed for the distance of the resonator edges from the surrounding coplanar ground cannot always be achieved immediately. A simple λ/4 transformer has been used to account for the operative frequencies of the designed resonators to address the signal and match the device adequately at the output ports of the SPDT. Individual resonators have been optimized by varying the distance between the triangle and the microstrip line, and it has been found that the optimal distance for the best coupling conditions depends on the internal complexity of the Sierpinski configurations, once the substrate’s geometry and material properties are chosen. Simple analytical derivations of the resonance frequency agree with the simulated responses of the whole triangles, and a trend of resonance for the Sierpinski triangles is a function of internal complexity. Finally, good electrical performances are predicted for the studied structures, including the ideal SPDT layout. It might be evidenced that the shape modification of a triangle can also efficiently modify the resonant frequency, e.g., by cutting corners or even scaling the original one. Nevertheless, because of the possible utilization of the Sierpinski triangles in complex arrangements, including arrays of triangles equal between them or exhibiting different internal complexity but having the same edge size, the modeling of the coupling mechanisms is facilitated. This allows the provision of a frequency selection using the same elementary cell. As discussed in the paper, it must be stressed that the increased internal complexity makes it difficult to predict the electrical response because the metal configuration in front of the microstrip is drastically modified, and it has consequences for the coupling, too. For this reason, we preferred to change only one crucial parameter, i.e., the Sierpinski complexity, always bearing in mind its possible utilization in arrays of coupled structures. It is not to be neglected that even triangles characterized by a different internal complexity can be coupled.

Concerning the utilization of such devices and, in particular, the connection of resonators using RF MEMS switches, this is now well understood in scientific literature. The novelty of this work lies in testing a building block for reconfiguring subsystems. The necessity of doing this is well documented, and we have included a few papers in the reference list to provide examples of the envisaged applications. The possibility of switching between different resonators enables frequency selection, which is particularly useful for multi-band, reconfigurable antenna systems and oscillators [11,12,13]. Nevertheless, a fully passive frequency selector, utilizing RF MEMS devices to select the operating frequency, is a significant advantage, as previously emphasized, due to lower losses and the absence of semiconductor devices that contribute to noise and nonlinear responses.

All the simulations have been performed using Microwave Office (MWO) Axiem, release 17.

## 2. Microstrip Design of Single Sierpinski Resonators

Triangular Sierpinski resonators have been studied using a 50-ohm microstrip transmission line as the feeding line and searching for an optimal response of the notch filter corresponding to the best electrical matching of the resonator. In particular, the distance between the transmission line and the resonator has been changed to optimize the coupling. The substrate chosen for the simulations is a high-resistivity silicon (HRS) wafer that is oxidized on both sides. Considering the matching for the resonators feeding line, a 50-ohm line is obtained by imposing a 515 µm wide microstrip for a grounded 640 µm thick HRS. The high-resistivity silicon (HRS) wafer used for the simulation has the characteristics of a commercially available substrate with a thickness of 640 μm, resistivity higher than 15,000 ohm-cm, and dielectric constant (permittivity) ε_Si_ = 11.9. The wafer is thermally annealed with a 1 μm thick layer of silicon oxide, i.e., the typical thermal oxide thickness used before processing the substrate with patterning of the metal structures on one side, with a dielectric constant of ε_SiO2_ = 3.9. Using the static approach of the TXLine utility implemented in the Cadence AWR Microwave Office, a width of 515 μm has been obtained for a 50-ohm microstrip line at 10 GHz.

After that, 6000 μm edge long triangles with internal complexity from 0 to 3, i.e., Sierpinski structures with internal triangles ranging from 1 (entire shape without holes) to 27 (the third iteration in the internal subdivision), have been designed, with a grating of 200 μm separating each sub-element of the structure. The Sierpinski triangles are figures belonging to the more general group of fractals. They are obtained by a progressive subdivision of whole triangles into many internal triangles of decreasing size, as discussed in [14,15,16].

The number of internal triangles is easily obtained by a law N_t_ = 3^n^, where “n” is the internal complexity index. It must be noticed that a growing number of internal triangles corresponds to a vanishing area and an increasing perimeter for the entire figure [17]. The four triangles chosen for the present study have been defined from C0 to C3, depending on the level of complexity C.

The optimization has been performed by searching for the optimal distance between the resonator and microstrip, which is different for each shape, looking at the deepest peak in the two-port band-stop arrangement. This difference has been interpreted owing to the shape difference between the triangle edge and the microstrip transmission line, which is homogeneous and continuous only in the case of the C0 shape. Still, it is fragmented for C1, C2, and C3, significantly modifying the boundaries involved in the coupling, owing to the discontinuities introduced by a growing number of sub-triangles.

The symmetric configuration, with two triangles having the same geometry on both sides of the microstrip, guarantees a deeper peak, thus increasing the quality factor of the resonator and providing a more homogeneous distribution of the electromagnetic field around the entire structure, hence not allowing the generation of additional spurious modes close to the main resonance in the spectral response of the resonators, as has also been discussed in [8]. A symmetric configuration is characterized by a deeper peak, better coupling, and quality factor, as already demonstrated in the study of the CPW-based resonators in [8] because the power brought by the transmission line (either the central conductor of the CPW or a microstrip) is homogeneously distributed on both sides of the feeding line. It is equivalent to considering two resonators critically coupled with the line, thus enhancing the power transferred to the entire resonating structure.

**Figure 1:**
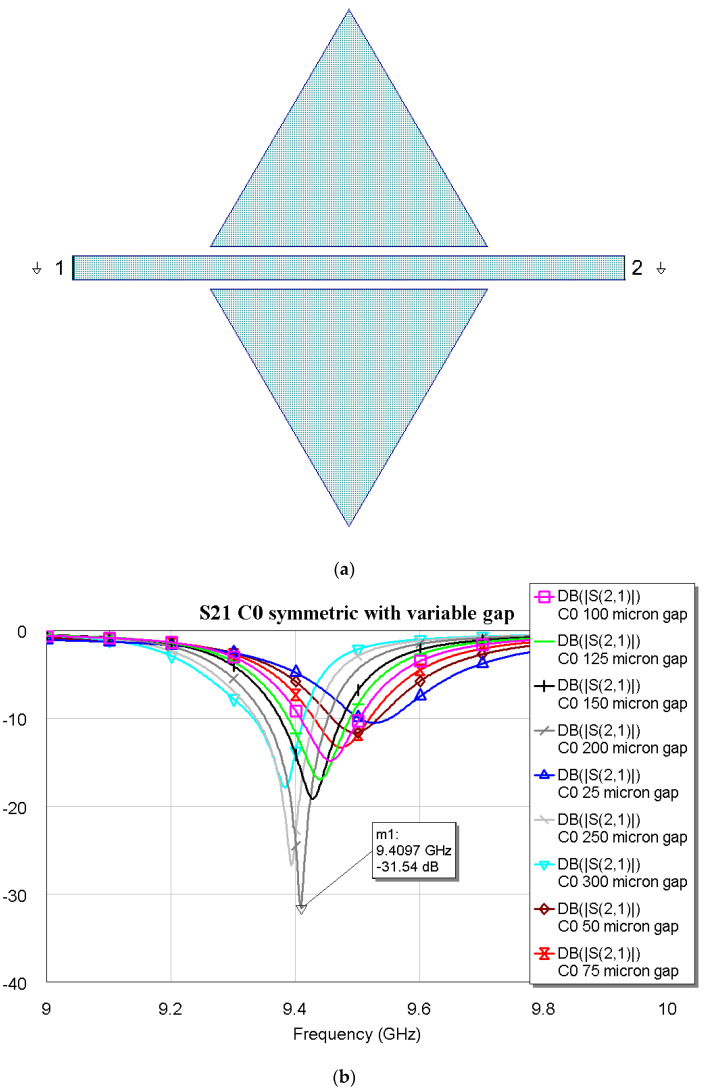
(**a**) The resonator layout for C0 configuration, and (**b**) the transmission coefficient S21 obtained from electromagnetic simulation using MWO.

Figure 1a,b present the resonator layout for the C0 configuration and the transmission coefficient S21 obtained after the structure was simulated using Cadence AWR Microwave Office. The triangle has an edge that is *a* = 6 mm long, while the microstrip exceeds the resonator size by 3 mm on each side.

The ground symbol close to the I/O ports of the configuration in Figure 1a means that the software considers the metalized bottom of the wafer as the reference ground for the simulated structure. Like all the other figures in the paper, this symbol has the same meaning.

The simulations shown in Figure 1b and those in the following figures have been performed using a cell with 10 μm resolution and the Axiem environment implemented in Microwave Office software. The Cadence^®^ AXIEM^®^ 3D planar EM solver is “a Method of Moments solver that solves for the currents on conductors that can be embedded in a stack of planar dielectric layers” [18]. The dielectric layers are infinitely extended in the x-y plane. They are defined above an infinite half-space. The half-space below the dielectric is typically a conductor or PEC (perfect electric conductor), but it can also be an infinite open boundary if needed. The half space above the dielectric layers is typically an infinite open boundary that correctly models free-space radiation, but it can also be a conducting plane. In our simulations, the material characteristics have been imposed to obtain as accurate as possible predictions of the configurations to be manufactured. Then, the shape and losses of the resonance peak are due to metal conductivity (Au, σ = 4.1 × 10^7^ S/m) and thickness (5 μm, considering the electroplating), tanδ = 0.002 for Si and SiO_2_, and the efficiency of the electrical coupling between the feeding line and the resonator. Fixing the material properties, the notch depth is determined by the coupling efficiency, mainly due to the optimal distance between the feeding line and the resonator.

**Figure 2:**
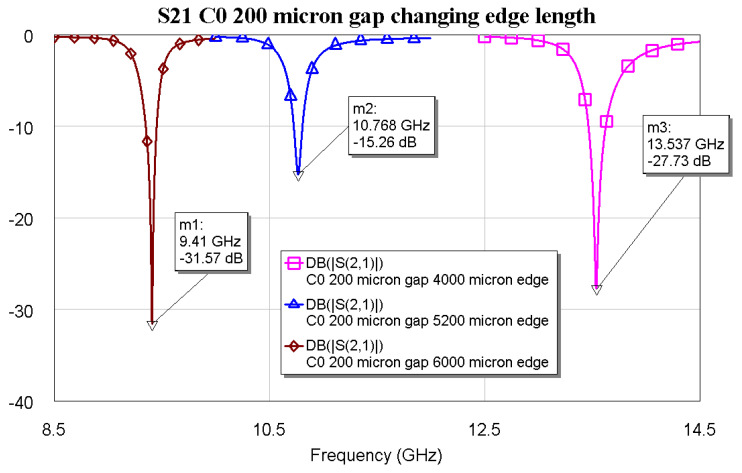
The transmission coefficient S21 resulting from C0 configuration with variable length was obtained by the electromagnetic simulation of the structures using Cadence AWR Microwave Office.

It is evident from Figure 1b that the deepest peak is obtained by imposing a distance of 200 µm between the microstrip and the two symmetric triangles. The C0 configuration has also been studied by changing the side length of the triangle and fixing the distance with respect to the microstrip, while also maintaining the I/O lines at a length of 3 mm. This procedure is another way to obtain different resonance frequencies, which is well established from the resonators and patch antenna theory. It has to be noticed that good electrical matching is expected at least until the beginning of the K-Band. Additional simulations are needed to confirm that the same boundary geometric conditions can be extended to higher operative frequencies. The results of the preliminary simulations for possible K-Band extension are shown in Figure 2 for three triangular symmetric C0 configurations, with edge lengths of *a* = 6000, 5200, and 4000 µm, respectively.

**Figure 3:**
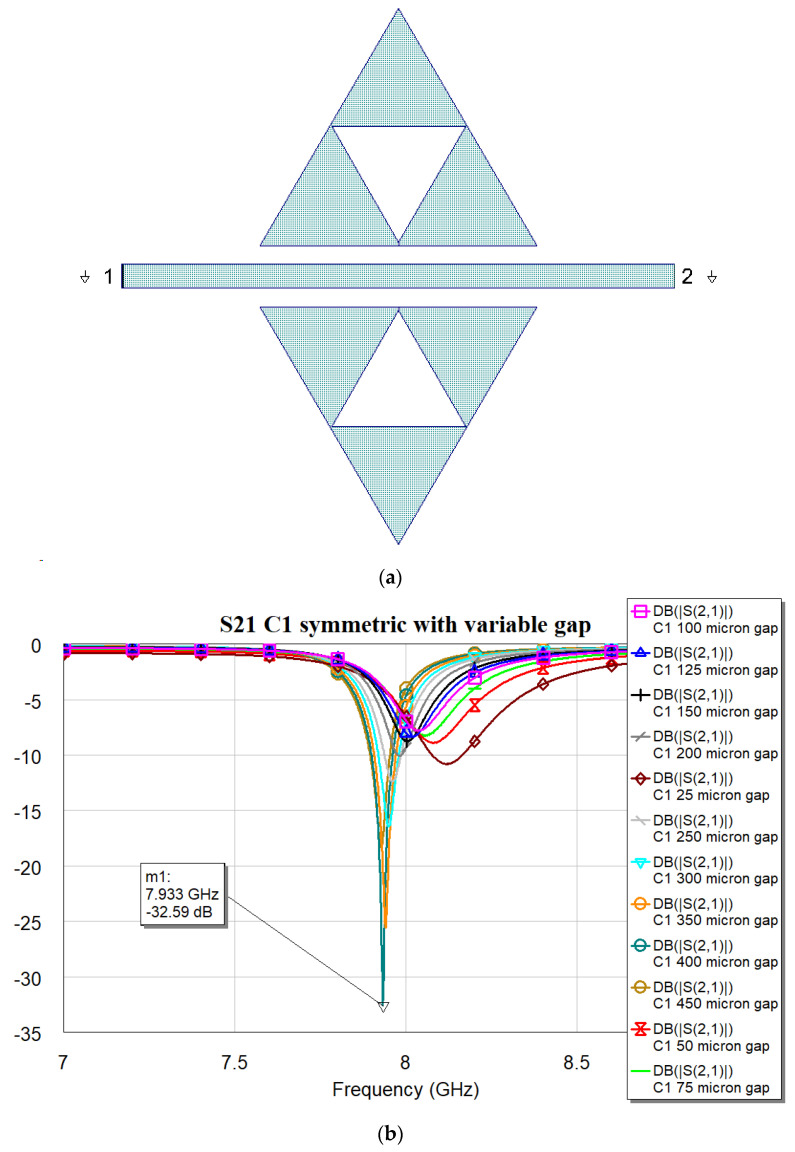
(**a**) The resonator layout for C1 configuration; (**b**) transmission coefficient S21 obtained by electromagnetic simulation using MWO for different distances between the triangles and microstrip line.

**Figure 4:**
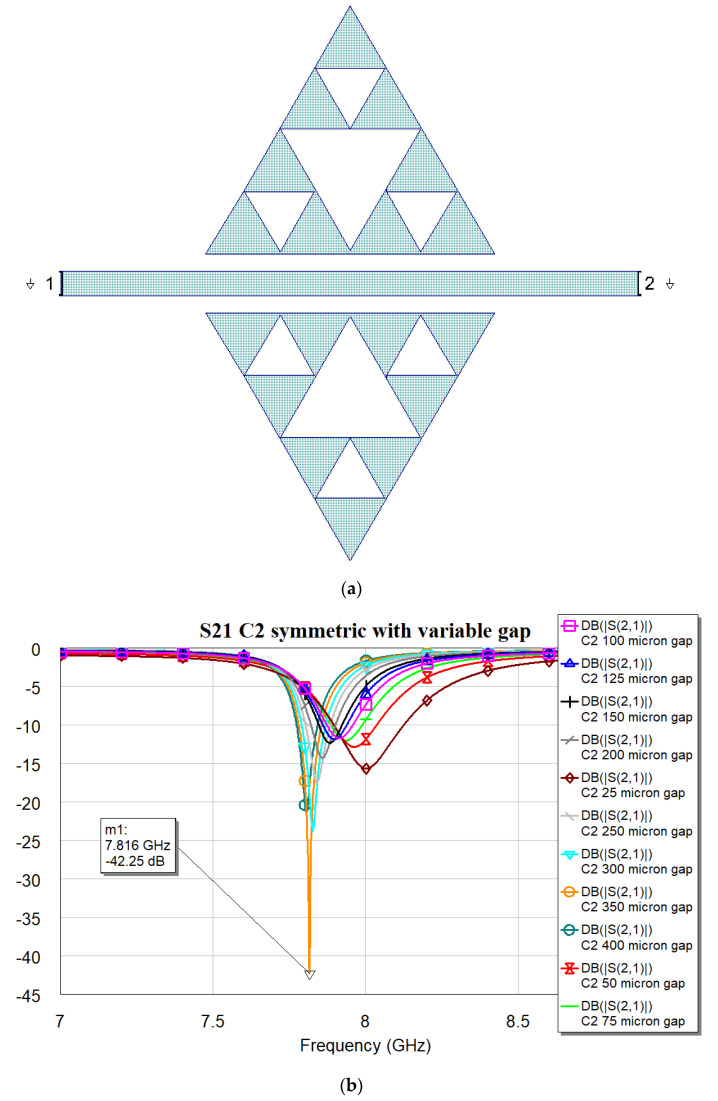
(**a**) The resonator layout for C2 configuration; (**b**) transmission coefficient S21 obtained by electromagnetic simulation using MWO for different distances between the triangles and microstrip line.

**Figure 5:**
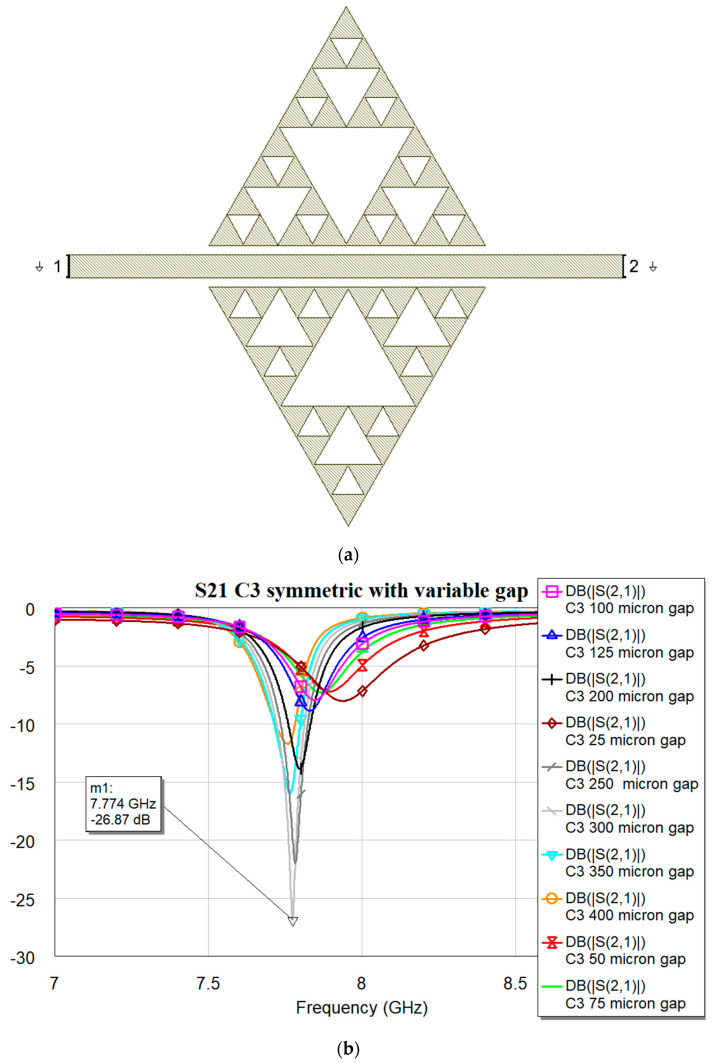
(**a**) The resonator layout for C3 configuration; (**b**) transmission coefficient S21 obtained by electromagnetic simulation using MWO for different distances between the triangles and microstrip line.

This preliminary evaluation is necessary to confirm the agreement between the original analytical approach to determine the resonance frequency, also according to experimental results, and the performed electromagnetic simulation. The equation governing the resonance frequency of a triangular metallic shape on a substrate with an dielectric constant ε was studied in detail and utilized in [9,19], and is given as follows:

(1) $$f_{resonance}=\frac{2c}{3a\sqrt{\varepsilon }}$$

$${a\to a}_{effective}=a+\frac{h}{\sqrt{\varepsilon }},\varepsilon \;unchanged$$

$$f_{resonance,effective}=\frac{2c}{3a_{effective}\sqrt{\varepsilon }}$$

where *c* = light speed, ε = dielectric constant, *a* = edge length, and *h* = substrate thickness. It is well known from the seminal works [9,19] that only the edge length must be corrected by an effective value, while the dielectric constant is the actual one of the substrate, as shown in Equation (1), which results in an effective resonance frequency value. A more detailed calculation was also performed in [20]. The comparison among the resonance frequencies is shown in Table 1, where very good agreement is obtained using both approaches. The same method has been used for triangles with higher complexity, and the shape and the simulation results are shown in Figure 3, Figure 4 and Figure 5, respectively, for the main mode. The spectrum, including higher-order modes, was calculated in [9].

**Figure 6:**
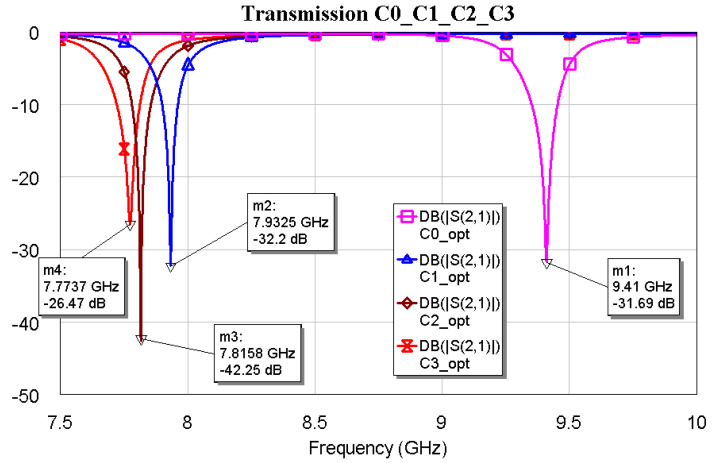
The transmission coefficient S21 was obtained by the electromagnetic simulations for the optimized (abbreviated “opt”) individual Sierpinski resonators, each separated from the microstrip feeding line by the optimal distance corresponding to the deepest peak.

**Figure 7:**
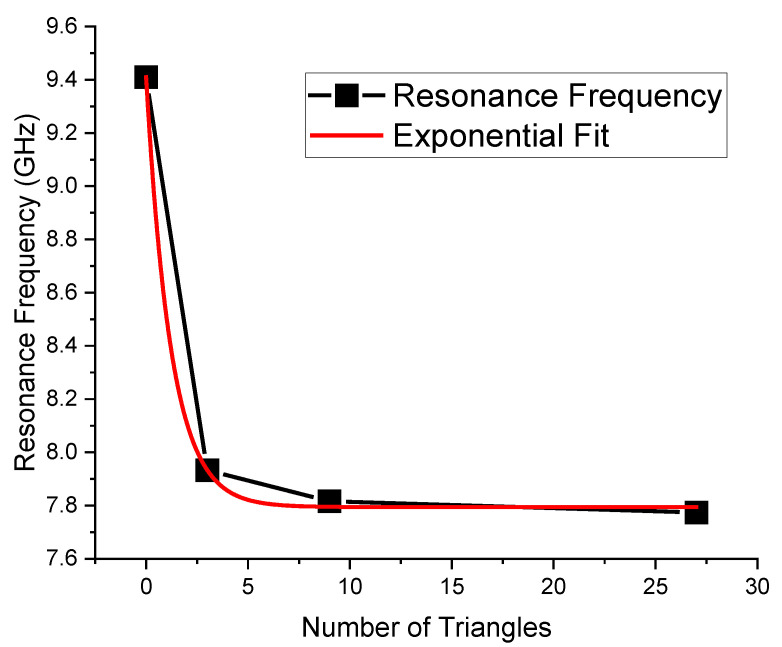
Electromagnetic simulated resonance frequency response for the optimized individual Sierpinski resonators as a function of the number of triangles N created inside the single triangle and exponential fit.

It is worth noting that the optimal matching is obtained for a distance close to the microstrip when the complexity is “0”, while it exhibits an increase and a slow decrease for C1, C2, and C3 with values of 400, 350 and 300 μm, respectively. The “jump” could be justified by the sudden change in the amount of metal when C1 is excited, with a single big triangular hole. At the same time, the increase in complexity also generates an increase in the number of sub-triangles close to the microstrip, with an increasing superposition with the electromagnetic field near the resonators. The predicted resonance frequencies for the optimal resonators are collected in the following plot (Figure 6), where it is evident the role that will be played by resonators having the same footprint but different complexity: C0 mixed with C1, C2 or C3 will generate a dual-band operation with frequencies that are quite far from each other, while an interplay using C1, C2, and C3 is better for fine-tuning. The trend of the resonance frequency as a function of complexity is shown in Figure 7.

Of course, we can obtain similar results by changing the resonator size. Still, it is more convenient to maintain a fixed footprint of the triangular patch, considering the possibility of combining the same elementary cell in more complicated filtering structures or antenna arrays.

The loaded quality factor of the resonators has been calculated using the usual definition of the resonance frequency in a ratio to the 3 dB bandwidth, i.e., Q_L_ = F_resonance_/BW, where BW is the 3 dB bandwidth. It is given in the following Table 2.

At the very beginning, one can imagine that the resonators with increasing complexity can be considered a kind of perturbation for the full triangular patch. Still, the internal complexity of the Sierpinski figure also modifies the metal configuration in front of the feeding line. This specific aspect, namely the interface between the Sierpinski triangle and the transmission line, modifies the electromagnetic field locally, thus impeding a homogeneous interface and excitation by the microstrip.

For the above reason, even in optimal conditions, there are some differences among the peak depths, bandwidths, and quality factors. Nevertheless, based on the numerical results in Table 2, the change can be considered negligible.

Since the triangular shape is preserved, and only the internal complexity is changed, Equation (1) can be modified by accounting for the trend shown in Figure 7, fitting the curve as a function of the internal number of sub-triangles.

The result in Figure 7 has been fitted as a possible modification of Equation (1). In this case, the following equation can be written:

$$f_{resonance,effective}=1.61*\exp\left(-\frac{N}{1.22}\right)+7.79\;\;\;[GHz]$$

On the other hand, this fit hides several geometric parameters, and it can be generalized only for the current geometry, which includes the frame thickness (in this case, the grating has a thickness of 200 μm, separating the individual sub-triangles) and the edge external length (6000 μm).

## 3. Simulation of the Dual-Band Sierpinski Resonators Driven by the RF MEMS SPDT

A single-pole-double-thru (SPDT) switch has already been used in the study of coplanar waveguide (CPW) resonating structures made by Sierpinski triangles to select two possible frequencies corresponding to two different resonators placed at the output of ports 2 and 3 of the SPDT, fed by a microwave signal coming from the port 1. The SPDT was designed and manufactured using microsystem technology and RF MEMS switches to obtain the desired port for the output signal. In the previous case, the bridges were ohmic structures in a CPW environment, causing the passage of the high-frequency signal when a suspended metal beam is actuated using an electrostatic force provided by a DC signal feeding the switch to cause its collapse for a threshold voltage [6]. In this case, the mechanical functionality and the kind of electrical contact remain unchanged, but the lateral ground planes have been removed, and the structure has been transformed into a microstrip configuration. The geometry and technology for the SPDT device’s manufacturing process are described in [6], which provides all the details of the 3D configurations. Simulations based on the method of moments are using a 2.5D environment [15]. Nevertheless, in our experience during past contracts for RF MEMS reliability, 2.5D simulations with correct material parameters and planar beams simulated in the up and down position with a 3 μm distance from the wafer surface fulfill the experimental response of the MEMS switch well. For this reason, a 3D simulation does not add more information to the prediction of the electrical response of the RF MEMS switch.

**Figure 8:**
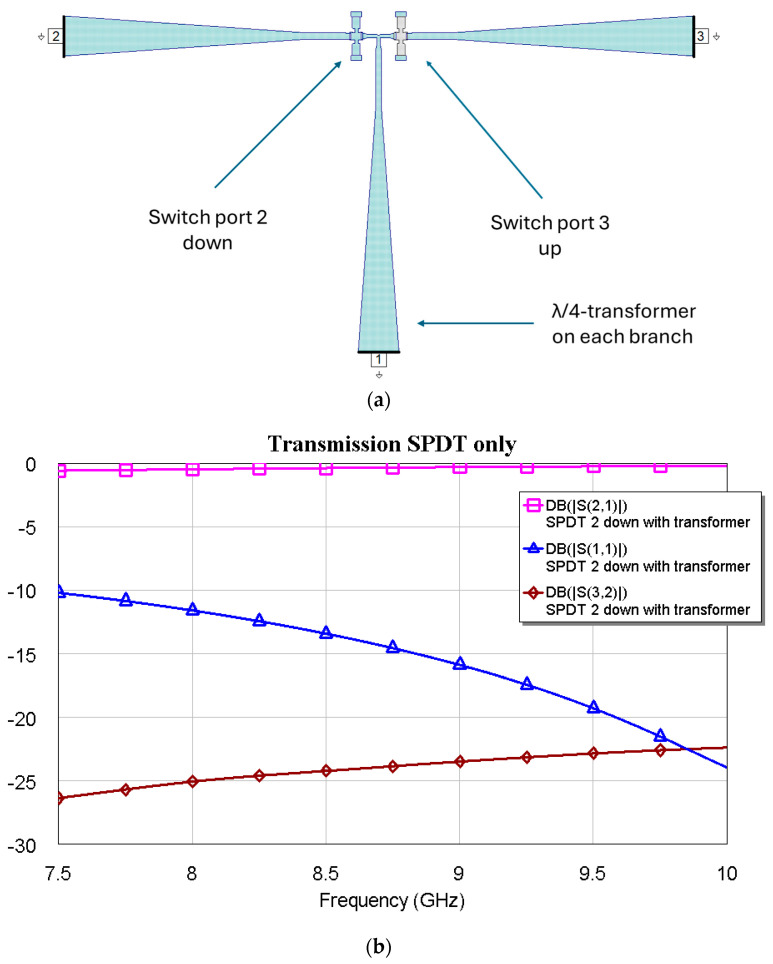
(**a**) SPDT Configuration, and (**b**) electromagnetic simulation performance (S21 = transmission parameter, in dB), S11 = reflection, and S32 = isolation) for the SPDT switch with the RF MEMS on port 2 actuated, thus providing the passage of the signal on port 2 and leaving port 3 isolated. The same performance is obtained when the switch on port 3 is actuated.

Considering the predicted frequencies of operation for the resonators to be connected, a simple λ/4 transformer has been used, working primarily between 7.5 GHz and 9.5 GHz. Even in a 2 GHz frequency range, the SPDT exhibits a reasonable response, which is helpful for a low-loss device within the X-Band. The configuration includes two switches and three arms with a transformer for each port, and its configuration and electrical performance for the output at port 2 are shown in Figure 8, when the switch before port 2 is actuated. The metal beam of the switch at port 3 is in the up position, providing the isolation for port 3. The symmetry of the structure guarantees the same result when the switch on port 3 is actuated (down position of the micromechanical bridge).

After optimizing the individual resonators and matching the SPDT in the X-Band, they were all connected to obtain a frequency selector working on a dual-band base. In particular, C0 was used with C1, C2, and C3 for tunability over an extended band, whereas C1, C2, and C3 were connected to achieve tunability over a small bandwidth. The results are shown in the following figures. The interconnection marginally changed the resonance frequencies, demonstrating the feasibility of a simple matching network to ensure the entire device is working correctly.

**Figure 9:**
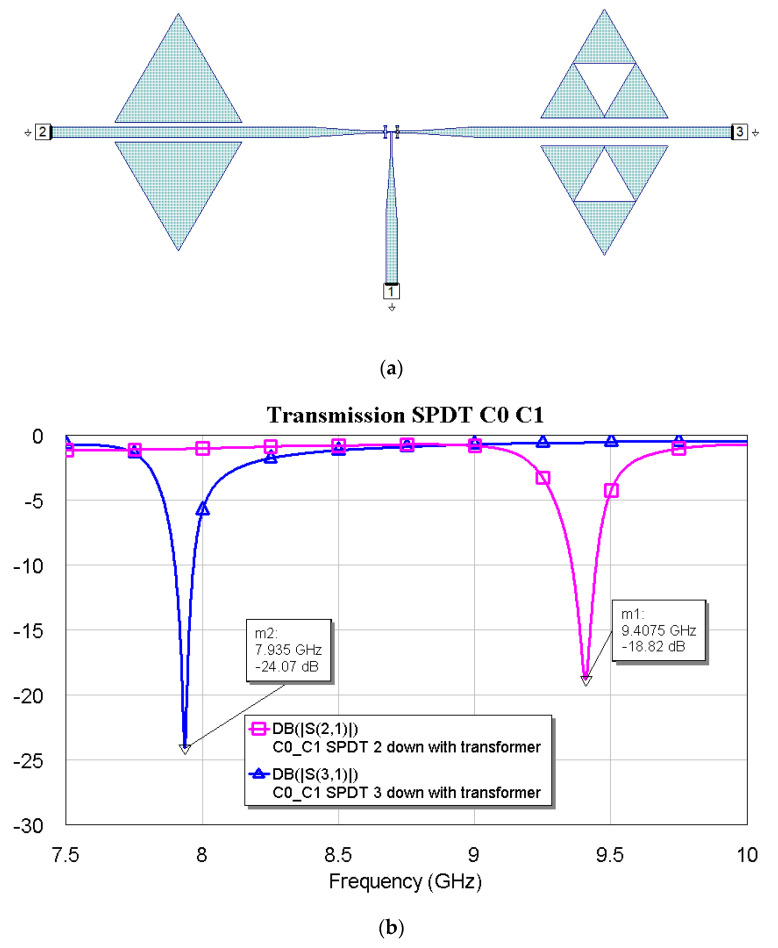
(**a**) SPDT connected with C0 and C1, and (**b**) simulated response for the transmission depending on the actuated branch of the SPDT (2 or 3).

**Figure 10:**
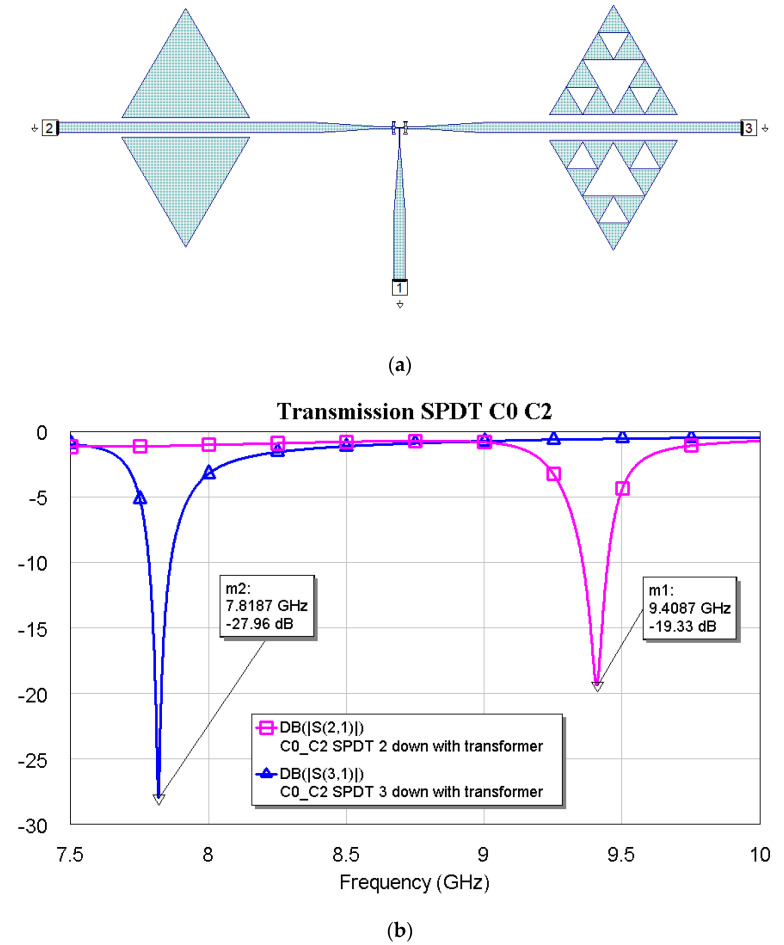
(**a**) SPDT connected with C0 and C2, and (**b**) simulated response for the transmission depending on the actuated branch of the SPDT (2 or 3).

**Figure 11:**
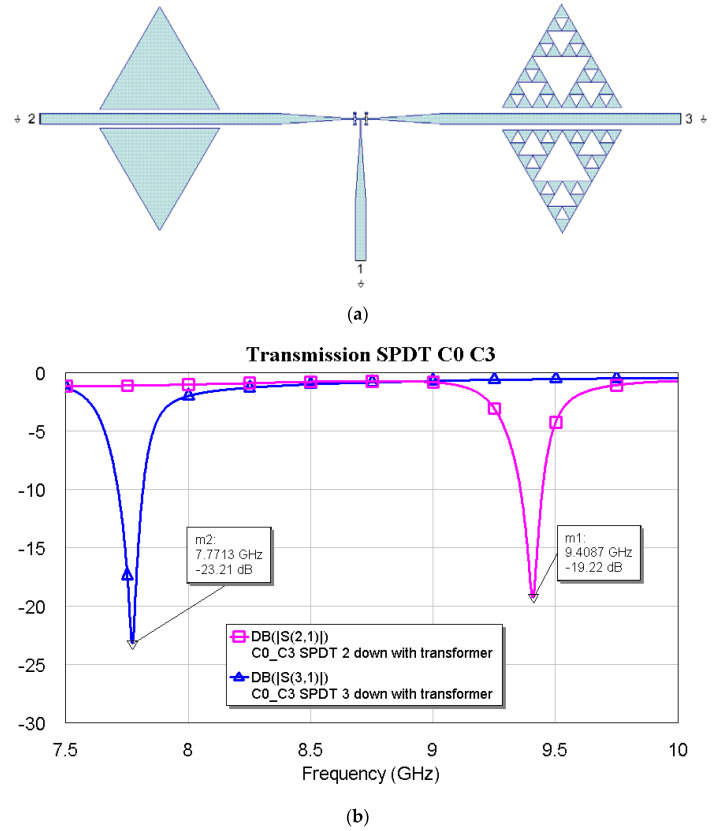
(**a**) SPDT connected with C0 and C3, and (**b**) simulated response for the transmission depending on the actuated branch of the SPDT (2 or 3).

Figure 9, Figure 10 and Figure 11 show the interconnections between C0 and the other three resonators with the simulated responses.

**Figure 12:**
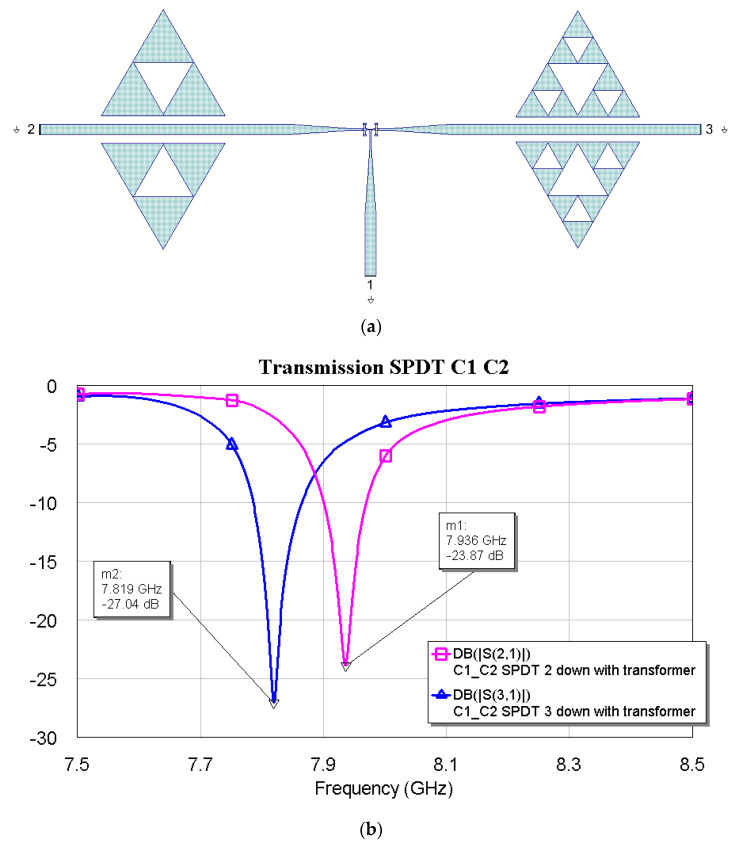
(**a**) SPDT connected with C1 and C2, and (**b**) simulated response in a narrow band for the transmission, depending on the actuated branch of the SPDT (2 or 3).

**Figure 13:**
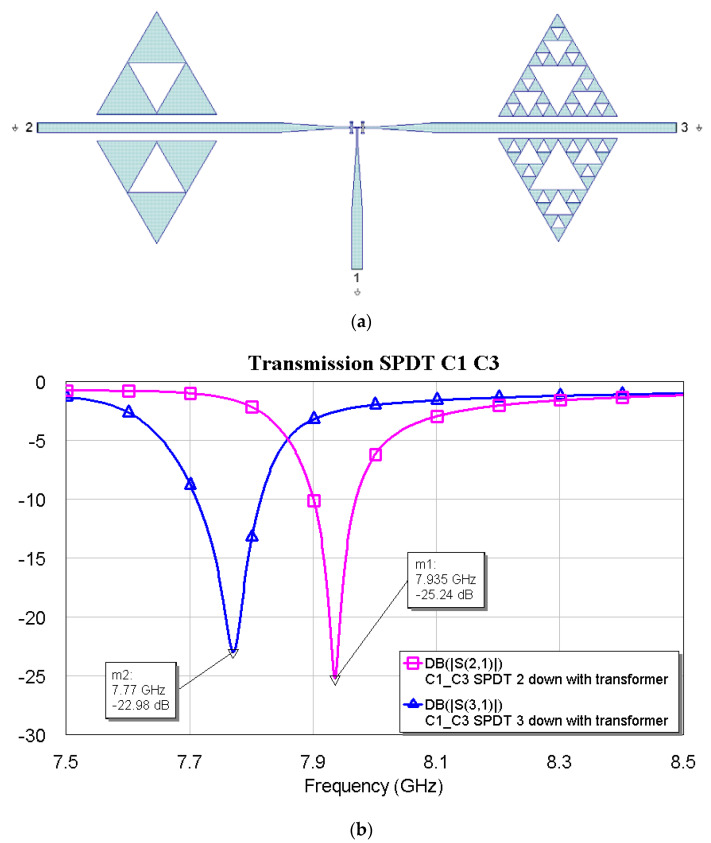
(**a**) SPDT connected with C1 and C3, and (**b**) simulated response in a narrow band for the transmission, depending on the actuated branch of the SPDT (2 or 3).

**Figure 14:**
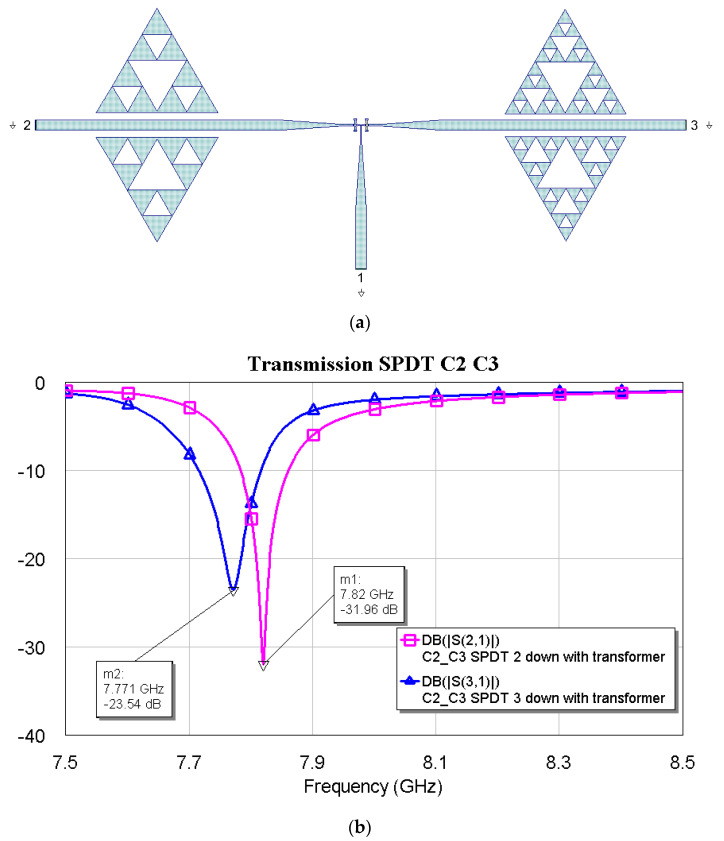
(**a**) SPDT is connected with C2 and C3, and (**b**) a simulated response in a narrow band for transmission, depending on the actuated branch of the SPDT (2 or 3).

The above plots show a good response for selecting frequencies at approximately 2 GHz between them. Fine-tuning has been studied using the couples C1–C2, C1–C3, and C2–C3, and the simulations are plotted in Figure 12, Figure 13 and Figure 14.

The above results show that the tuning becomes finer and finer as the internal complexity of the Sierpinski resonator increases. This result was also expected based on the individual responses of the resonators studied before. No criticalities have been observed when it comes to achieving a good electrical response in both situations, with excellent loss levels and peak depths of the notch filters.

## 4. Experimental Results

A few structures have been manufactured to confirm the validity of the design approach described in this paper. In particular, the configurations C0 and C1 have been studied as a single resonator and combined by using the ideal SPDT, without actual RF MEMS, but emulating, like in the case of the simulations presented in the previous paragraphs, the states “open” and “closed”, i.e., the switch states corresponding to the membranes up or down (“off” and “on” from the switch functionality point of view). The ideal SPDT used in this experiment is not a commercial product, but a configuration manufactured to emulate the “on” and “off” functionality of a real MEMS SPDT. As established, the actual MEMS is a metal beam that can be electrostatically collapsed by applying a DC voltage. In the “up” position, the actual beam is distant 3 μm from the surface of the wafer, thus guaranteeing the best experimental electrical isolation and an “open” condition. For this reason, as was already the case in past contracts for preliminary evaluations, the ideal “open” is equivalent to removing the metal beam, leaving the microstrip with a gap (which is filled in the actual case when the bridge collapses). The same consideration has to be made for the “on” electrical state of the switch. The ideal case corresponds to a continuous line, without interruptions. In our ideal configuration, the metal is deposited continuously to emulate the closure of the MEMS switch. The above solution should be considered the best obtainable experimental condition. The fabrication of a device with the ideal SPDT is used for fast (only one mask process) and cheap experimental verification of the designed devices. It allows a further optimization before the fabrication of real devices with miscroswitches that is longer (8 mask fabrication process) and much more expensive. In particular, six preliminary configurations have been manufactured and tested, including (i) C0 and C1 single resonators with two distances of the resonator from the feeding microstrip (50 microns and optimized distance; four structures); (ii) SPDT linked to C0 and C1 in the on and off positions (two structures). The technology used to realize the tested devices is a one-mask photolithographic process performed on a thermally oxidized, high-resistivity silicon substrate. On the front, 5 µm thick patterned Au has been grown by electroplating, while on the backside, 1.2 µm thick Al has been sputtered as ground.

**Figure 15:**
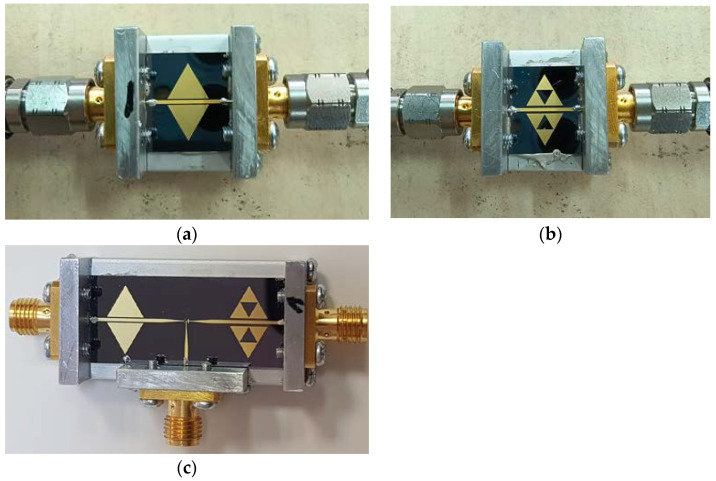
Experimentally tested devices: (**a**) C0 configuration; (**b**) C1 configuration; (**c**) entire device with ideal SPDT, tapered lines to provide the impedance transformation in the studied frequency range, connected to C0 and C1 at the output ports of the three-port structure.

Figure 15a (C0 configuration), Figure 15b (C1 configuration), and Figure 15c (the entire device, including SPDT connected to C0 and C1) show the manufactured structures, with the connectors used for testing them by a vector network analyzer characterization.

**Figure 16:**
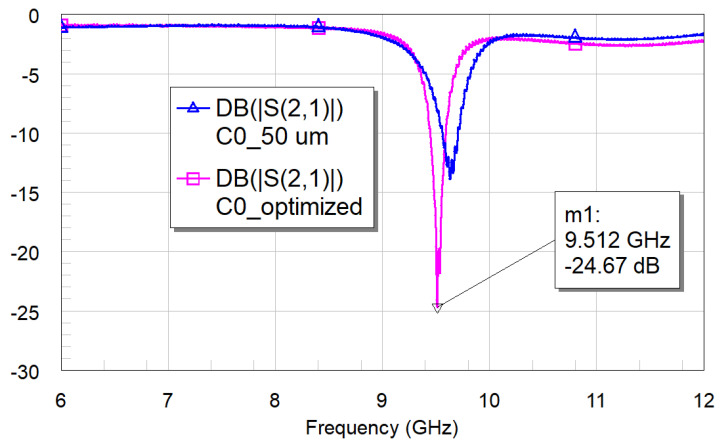
Experimentally tested C0 configuration, using a 50 μm distance to the feeding line, and optimizing the distance (200 μm).

**Figure 17:**
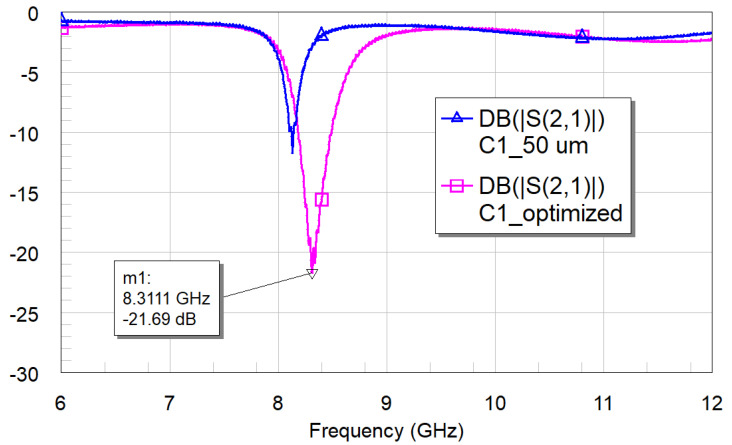
Experimentally tested C1 configuration, using a 50 μm distance to the feeding line, and optimizing the distance (300 μm).

The measurements shown in Figure 16 for the resonator C0 and Figure 17 for the C1 geometry demonstrate the necessity of optimizing the distance between the resonator and the feeding line to achieve the best coupling conditions.

Parasitic contributions can modify the frequency of resonance, which is 9.515 GHz, vs. the predicted value of 9.410 GHz obtained from the simulations for C0. Still, the peak amplitude agrees with the simulated one, being −29.15 dB compared to the value of −31.54 dB for the optimized resonator, as shown in the simulation presented in Figure 1.

The same can be said about the C1 configuration, for which the measured resonance frequency of the optimized structure is 8.32 GHz vs. the simulated 7.933 GHz. In this case, the measured peak also disagrees with the simulation, even if its amplitude guarantees a suitable electrical coupling with an experimental value lower than −20 dB.

**Figure 18:**
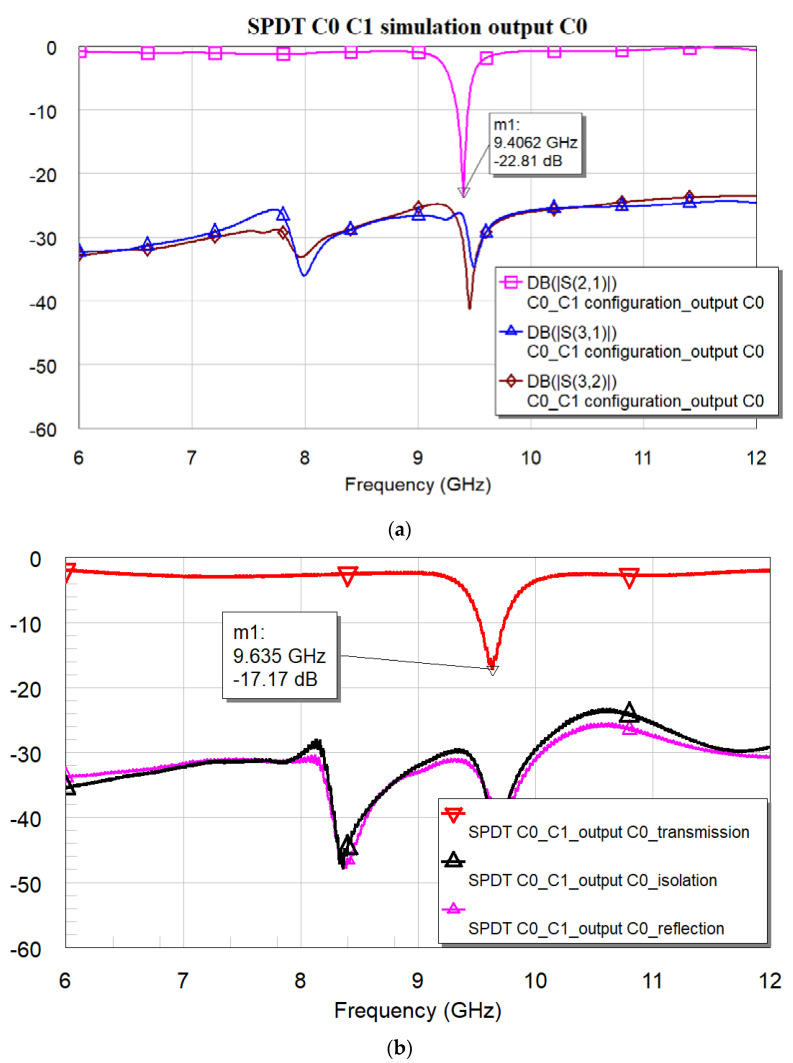
Expected response (**a**) and experimental results (**b**) for the entire three-port device comprising C0, C1, SPDT, and transformer. Output on port 2, where the C0 resonator is located.

**Figure 19:**
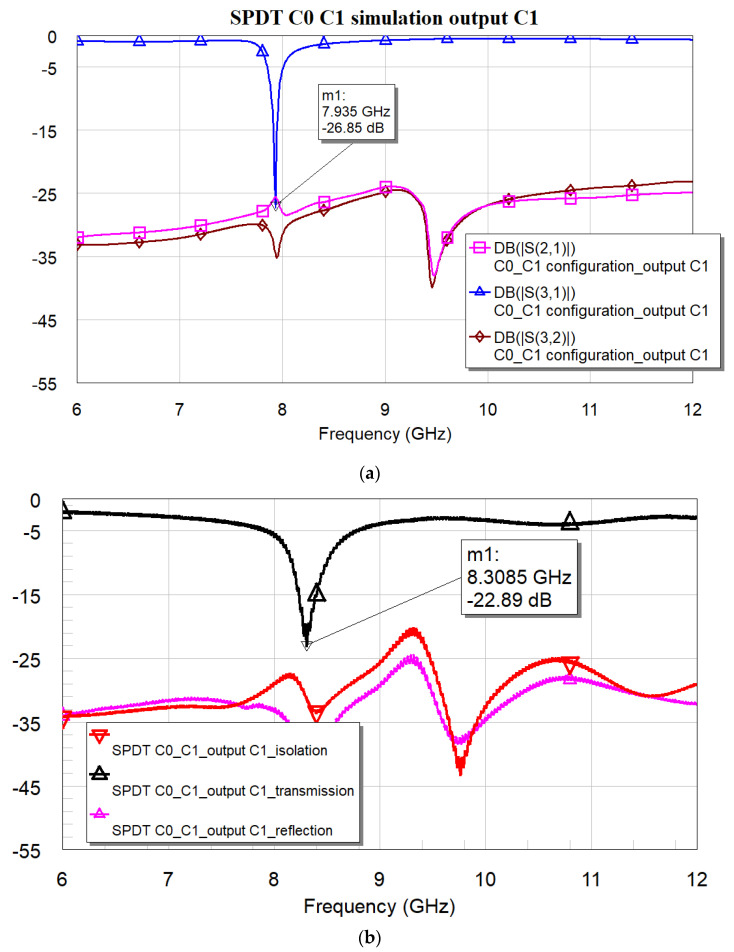
Simulation (**a**) and experimental results (**b**) for the three-port device comprising C0, C1, SPDT, and transformer. Output on port 3, where the C1 resonator is located.

Figure 18a,b present the expected and measured performance for the three-port device, comprising C0 and C1, when the C0 branch is activated (output on port 2, selecting C0). Figure 19a,b show the predicted and measured responses for the same configuration, where the C1 branch is now “on” and the signal is transmitted through port 3. Figure 18 and Figure 19 have been arranged to avoid overlap between the theoretical and experimental curves, ensuring clarity throughout the presentation. However, markers have been used to indicate the selected resonance frequency and the amplitude of the corresponding S-parameter (S21 for port 2 and S31 for port 3).

The expected resonance for the C0 output was at 9.406 GHz and −22.81 dB, while the measured values are 9.627 GHz and −17.25 dB when port 2, i.e., C0, is activated.

For port 3, we have a predicted resonance frequency of 7.932 GHz with an amplitude of −32.59 dB, and measured values of 8.311 GHz and −21.69 dB when port 3, i.e., C1, is active.

It is worth noting that the overall electrical performance still requires some improvement, particularly regarding the parasitic contributions from the additional lines and the need for an optimized transformer, which, in this case, has been simplified for heuristic purposes. Parasitics or non-completely understood contributions, possibly arising from assembly, soldering, and connectors that have not been considered in the simulations, are also responsible for the frequency shift observed in the experiment compared to the simulations. On the other hand, the isolation data guarantee a good starting point for further improvements.

## 5. Conclusions

This paper proposes a micromechanical device for dual-band applications, utilizing triangular Sierpinski resonators as notch filters, which are selected using an RF MEMS-based SPDT switch. A microstrip configuration was chosen for the exploited structures. This study was performed with a complexity of the fractal triangles up to the third order, compatible with typical sizes of a classical photolithographic process. First, the electrical performance of the individual resonators was optimized for frequencies within the X-Band. Then, a λ/4 transformer was used to electrically match the three branches of the SPDT switch, connected to the optimized single resonators. The investigated structures exhibit interesting properties regarding the optimal distance between the resonator and the microstrip feeding line, as well as the possibility of interconnecting triangles with different Sierpinski complexities to achieve fine-tuning of the frequency. It is worth noting that in the case of resonators (or possibly patch antennas), the size could be a solution for selecting different frequencies, but maintaining the same footprint is vital considering the design and realization of filters and antenna arrays exhibiting the same shape and size but different internal complexity, thus providing frequency selection using the same elementary cell. Once the geometry is fixed, there are many possibilities for tuning the resonance frequency. Cutting a triangle’s corner to create a trapezoidal shape or adjusting the edge length are possible solutions. Nevertheless, using a fixed footprint is the precursor to eventually combining individual triangles into various arrangements, considering even the possibility of coupling resonators with the same internal complexity or a different one. A preliminary experimental verification was performed using the C0 and C1 resonators, demonstrating that the measured electrical performance was suitable for the proposed dual-band applications. Nevertheless, implementing details such as the electrical matching of the transformer and a more accurate determination of parasitic contributions, as well as actual details related to the device’s assembly, can improve the overall electrical response and better calibrate the desired resonance frequency.

## Figures and Tables

**Table 1:** Comparison between the resonance frequencies resulting from the electromagnetic simulation (EM) and those calculated analytically for the configuration C0 using Equation (1).

Edge Length (µm)	6000	5200	4000
F_resonance,analytical_ (GHz)	9.375	10.768	13.854
F_resonance,EM_ (GHz)	9.410	10.768	13.537

**Table 2:** Resonance frequencies and loaded quality factors for the four studied configurations (C0, C1, C2, and C3).

Configuration	Resonance Frequency [GHz]	3 dB Bandwidth [MHz]	Loaded Quality Factor Q_L_
C0	9.410	282	33.4
C1	7.932	217	36.6
C2	7.812	240	32.6
C3	7.774	259	30

## Data Availability

The original contributions presented in this study are included in the article. Further inquiries can be directed to the corresponding author.
